# Structural Interpretation of the Large Slowdown of Water Dynamics at Stacked Phospholipid Membranes for Decreasing Hydration Level: All-Atom Molecular Dynamics

**DOI:** 10.3390/ma9050319

**Published:** 2016-04-27

**Authors:** Carles Calero, H. Eugene Stanley, Giancarlo Franzese

**Affiliations:** 1 Secció de Física Estadística i Interdisciplinària—Departament de Física de la Matèria Condensada, Facultat de Física, Universitat de Barcelona, Martí i Franquès 1, Barcelona 08028, Spain; 2 Institut de Nanociència i Nanotecnologia, Universitat de Barcelona, Av. Joan XXIII S/N, Barcelona 08028, Spain; 3 Center for Polymer Studies and Department of Physics, Boston University, 590 Commonwealth Avenue, Boston, MA 02215, USA; hes@bu.edu

**Keywords:** water, molecular dynamics, confinement, phospholipid membrane, diffusion

## Abstract

Hydration water determines the stability and function of phospholipid membranes as well as the interaction of membranes with other molecules. Experiments and simulations have shown that water dynamics slows down dramatically as the hydration decreases, suggesting that the interfacial water that dominates the average dynamics at low hydration is slower than water away from the membrane. Here, based on all-atom molecular dynamics simulations, we provide an interpretation of the slowdown of interfacial water in terms of the structure and dynamics of water–water and water–lipid hydrogen bonds (HBs). We calculate the rotational and translational slowdown of the dynamics of water confined in stacked phospholipid membranes at different levels of hydration, from completely hydrated to poorly hydrated membranes. For all hydrations, we analyze the distribution of HBs and find that water–lipids HBs last longer than water–water HBs and that at low hydration most of the water is in the interior of the membrane. We also show that water–water HBs become more persistent as the hydration is lowered. We attribute this effect (i) to HBs between water molecules that form, in turn, persistent HBs with lipids; (ii) to the hindering of the H-bonding switching between water molecules due to the lower water density at the interface; and (iii) to the higher probability of water–lipid HBs as the hydration decreases. Our interpretation of the large dynamic slowdown in water under dehydration is potentially relevant in understanding membrane biophysics at different hydration levels.

## 1. Introduction

Phospholipid membranes provide the framework of biological membranes, which are ubiquitous in nature as the limiting structures of cells and organelles and separate interior contents from external environments. They are also the main component in the formation of liposomes, which are used as drug-delivery systems and in cosmetics [1].

Phospholipid membranes consist of two leaflets of amphiphilic lipids that self-assemble due to the hydrophobic effect [2]. The study of pure component membranes can help us understand how basic biological membranes function and how they interact with the environment. It is important to comprehend the properties of phospholipid membranes at different hydration levels because water regulates membrane stability and function and mediates themembrane interaction with solutes such as ions, proteins, DNA and other membranes [3].

A range of experimental techniques have been used to study the properties of water hydrating membranes and other biomolecules, including neutron scattering [4,5], nuclear magnetic resonance (NMR) spectroscopy [6,7], infrared spectroscopy [8], ultrafast vibrational spectroscopy [9], and terahertz spectroscopy [10]. In Ref. [7], the diffusion of water confined in the lamellar phase of egg phosphatidylcholine was investigated as a function of the hydration of the membrane. A significant reduction (of approximately a factor 10) of the water diffusion coefficient was reported for weakly hydrated systems and was attributed to the interaction with the membrane. Accordingly, the authors observe a monotonic increase in the diffusion coefficient with the membrane’s hydration. Recent experiments [5,9,10] have investigated the dynamical rotational properties of water molecules at phospholipid membranes with varying water content using different choline-based phospholipids and experimental techniques. The authors of these studies show a slowdown in the dynamics of water molecules at the membrane interface and distinguish different types of water according to its rotational dynamics: fast, irrotational, and bulk-like. They related their findings to the number of hydrogen bonds (HBs) formed by water molecules. All-atom molecular dynamics (MD) simulations have provided microscopic insights into the dynamics and structure of water at the interface with phospholipid membranes, demonstrating that water can be strongly bound via HBs to polar groups of the bilayer lipids [11,12]. This interaction was shown to restrict the dynamics of water resulting in longer reorientation times and slower translational dynamics [11,12,13]. MD simulations were successfully used to interpret experimental observations of the effect of the phospholipid membrane on the reorientation dynamics with different hydration levels and infrared spectra of interfacial water [14,15].

Here, we use all-atom MD simulations to study the diffusion and rotational dynamics of water hydrating stacked dimyristoylphosphatidylcholine (DMPC) phospholipid membranes. We vary the hydration level, *ω*, defined as the number of water molecules per phospholipid. Among a wide variety of lipids, DMPC are phospholipids incorporating a choline as a headgroup and a tailgroup formed by two myristoyl chains. Choline based phospholipids are ubiquitous in cell membranes and used in drug targeting liposomes [1]. Our findings relate the dynamical behavior of water confined between bilayers to the structure and dynamics of the HB network formed among water molecules and between water and selected lipid groups.

## 2. Results and Discussion

**Figure 1:**
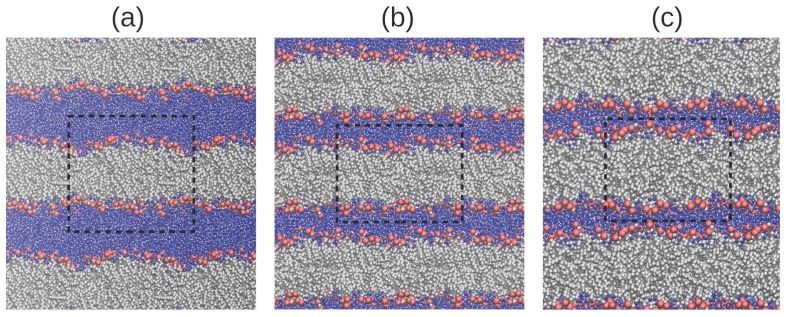

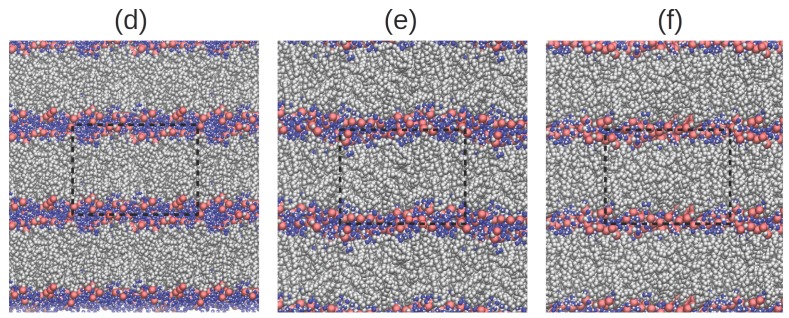
Snapshots of the six systems considered in our study, with hydration levels (**a**) 34; (**b**) 20; (**c**) 15; (**d**) 10; (**e**) 7; and (**f**) 4. Gray and red beads represent phospholipid tails and headgroups, respectively. Blue and white beads represent oxygen and hydrogen atoms of water. The dashed line indicates the size of the simulation box.

We perform MD simulations of TIP3P water confined between DMPC phospholipid membranes (see Methods section for further details). The use of periodic boundary conditions in our simulations allows us to describe a system of perfectly stacked phospholipid bilayers with a homogeneous prescribed hydration level *ω*. We consider six different hydration levels ω=4, 7, 10, 15, 20, and 34 (Figure 1), which include the low hydrated systems probed in experiment [8,9,10] and a fully hydrated membrane (with ω=34) [16].

### 2.1. Translational Dynamics

**Figure 2:**
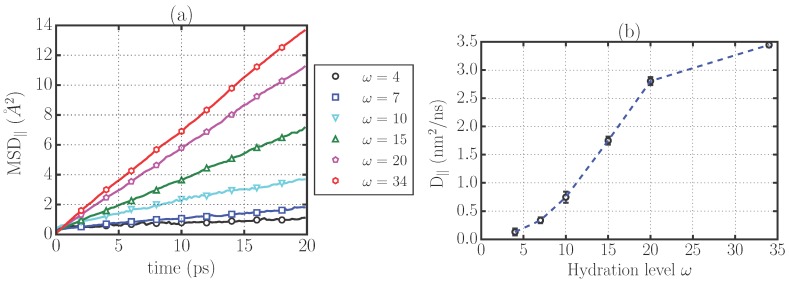
Translational dynamics of confined water molecules projected on the plane of the membrane for the different stacked phospholipid bilayers. (**a**) mean-square displacement on the plane of the membrane (MSD${}_{∥}$) as a function of time; (**b**) diffusion coefficient of water molecules on the plane of the membrane for the different hydration levels considered.

We characterize the translational dynamics of water confined in stacked DMPC bilayers by calculating the mean-square displacement of the molecule’s center of mass projected on the plane of the membrane (MSD${}_{∥}$). We simulate 10 ns-trajectories saved every 0.1 ps, discard the initial 2 ps until the diffusive regime is reached, and calculate the diffusion coefficients for confined water as:

(1) $$D_{∥}\equiv \lim_{t\to \infty }\frac{\langle |r_{∥}\left(t\right)-r_{∥}\left(0\right){|}^{2}\rangle }{4t},\;$$

where $r_{∥}\left(t\right)$ is the projection of the center of mass of a water molecule on the plane of the membrane and the angular brackets 〈...〉 indicate the average over all water molecules and time origins (Figure 2a).

We find (Figure 2) that the average over all the water molecules indicates a significant translational slowdown that is more pronounced as the hydration of the system is lowered, in agreement with previous experimental and computational studies [9,10,14,15]. Indeed, the diffusion coefficient averaged over the entire system is always much smaller than the bulk value $D^{\mathrm{bulk}}=5.5\pm 0.1$ nm${}^{2}$/ns and decreases monotonically for decreasing hydration (Figure 2b), from 3.4 nm${}^{2}$/ns for the completely hydrated membrane (ω=34) to $D_{∥}=0.13$ nm${}^{2}$/ns for the lowest hydrated system (ω=4). The strong dependence of $D_{∥}$ on hydration and its large drop at low *ω* are in qualitative agreement with experiments for similar systems [7,17]. Nevertheless, a quantitative comparison with experimental results might be problematic due to the difficulty in experiments to ensure the homogeneity of hydration and the perfect alignment of the membranes in measuring $D_{∥}$.

We finally observe that this result is consistent with recent theoretical advances in our understanding of the dimensionality-dependence of diffusion [18] if we consider that the membrane is generating a rugged energy landscape for the confined water. Indeed, the theory of diffusion in a rugged energy landscape shows that the reduction of the diffusion coefficient from 3d to 2d can be of an order comparable to our result (∼38% in our case).

### 2.2. Rotational Dynamics

**Figure 3:**
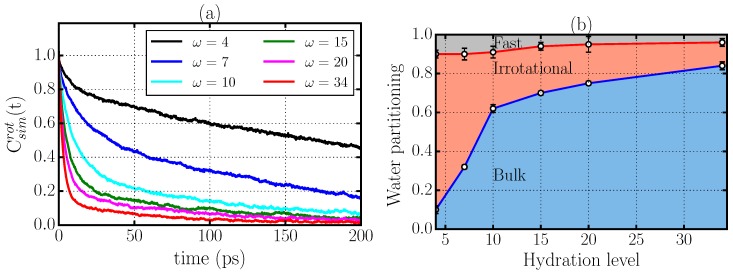
(**a**) rotational dipolar correlation function of water molecules calculated from simulations of DMPC phospholipid bilayers with different levels of hydration *ω*; (**b**) the partition of all the water in the system into the fractions (i) $f_{\mathrm{bulk}}$ of bulk-like (lower circles); (ii) $f_{\mathrm{irr}}$ of irrotational (upper circles) and (iii) $f_{\mathrm{fast}}=1-f_{\mathrm{bulk}}-f_{\mathrm{irr}}$ of fast water molecules, as a function of the hydration level *ω* in Equation (5), following the decomposition of the rotational correlation function proposed in Ref. [10]. The dots represent the partitioning from MD simulations. Full lines are guides for the eyes.

We next study the rotational dynamics of the water confined in stacked phospholipid bilayers. We calculate the rotational dipolar correlation function,

(2) $$C_{\mathrm{sim}}^{\mathrm{rot}}\left(t\right)\equiv \langle \hat{\mu }\left(t\right)\cdot \hat{\mu }\left(0\right)\rangle \;,$$

where $\hat{\mu }\left(t\right)$ is the direction of the water dipole vector at time *t* and 〈...〉 denotes the ensemble average over all water molecules and time origins (Figure 3a). This quantity is related to recent terahertz dielectric relaxation measurements used to probe the reorientation dynamics of water [10]. To quantify the relaxation of the correlation functions $C_{\mathrm{sim}}^{\mathrm{rot}}\left(t\right)$, we define the relaxation time:

(3) $$\tau_{\mathrm{rot}}\equiv \int_{0}^{\infty }C_{\mathrm{sim}}^{\mathrm{rot}}\left(t\right)dt\;,$$

which is not dependent on any assumptions about the functional form of the correlation function. The results for $C_{\mathrm{sim}}^{\mathrm{rot}}\left(t\right)$ and the corresponding relaxation times $\tau_{\mathrm{rot}}$ (Table 1) confirm the monotonic slowing-down of the rotational dynamics of water for decreasing membrane hydration as found in experiments and in previous computational works [9,10,14,15].

### 2.3. Criticism to the Interpretation of the Rotational Dynamics in Terms of Different Types of Water Molecules

An inspection of Figure 3 might suggest the existence of different time-scales in the decay of the rotational correlation function $C_{\mathrm{sim}}^{\mathrm{rot}}\left(t\right)$ for all the cases considered, *i.e.*, an initial rapid decrease of $C_{\mathrm{sim}}^{\mathrm{rot}}\left(t\right)$ followed by a much slower decay process. In the interpretation of dielectric relaxation experiments, Tielrooij *et al.* [10] assumed the existence of three different water species according to their reorientation dynamics, *i.e.*, (i) bulk-like water molecules whose reorientation dynamics resemble that of bulk water, with characteristic times $\tau_{\mathrm{bulk}}$ of a few picoseconds; (ii) fast water molecules that reorient significantly faster than bulk water with $\tau_{\mathrm{fast}}\approx$ fraction of picosecond; and (iii) irrotational water molecules that may relax with characteristic times $\tau_{\mathrm{irr}}\gg 10$ ps.

Here, we test the consistency of this hypothesis using our MD simulation results. We can identify the fractions $f_{\mathrm{fast}}$, $f_{\mathrm{bulk}}$ and $f_{\mathrm{irr}}$ of the three species as a function of *ω* by fitting $C_{\mathrm{sim}}^{\mathrm{rot}}\left(t,\omega \right)$ to a sum of pure exponentially decaying terms,

(4) $$C_{\mathrm{sim}}^{\mathrm{rot}}\left(t,\omega \right)=f_{\mathrm{fast}}\left(\omega \right)e^{-t/\tau_{\mathrm{fast}}}+f_{\mathrm{bulk}}\left(\omega \right)e^{-t/\tau_{\mathrm{bulk}}}+f_{\mathrm{irr}}\left(\omega \right)e^{-t/\tau_{\mathrm{irr}}},\;$$

where

(5) $$f_{\mathrm{fast}}\left(\omega \right)+f_{\mathrm{bulk}}\left(\omega \right)+f_{\mathrm{irr}}\left(\omega \right)=1$$

is the sum for each *ω* of the fractions $f_{\mathrm{fast}}$, $f_{\mathrm{bulk}}$, and $f_{\mathrm{irr}}$ of fast, bulk-like and irrotational water molecules, respectively (see Table 2 and Figure 3b).

Although Figure 3b qualitatively accounts for the experimental behavior reported in Ref. [10], the fitting procedure is not robust because there are five fitting parameters for each *ω*. In addition, the parameters $\tau_{\mathrm{fast}}$, $\tau_{\mathrm{bulk}}$, and $\tau_{\mathrm{irr}}$ in Table 2 are not showing any regular behavior as a function of *ω*. In particular, we would expect that $\tau_{\mathrm{bulk}}$ should be independent of *ω*, but instead it is varying non-monotonically by a factor >2.

All of these observations suggest that the interpretation of the dynamics slowdown proposed in Ref. [10] in terms of these distinctive types of water may not be complete, and that a more thorough analysis is needed. In the following, we propose an interpretation based on an analysis of the structure and dynamics of the HBs.

### 2.4. Hydrogen Bond Structure

In order to understand the results obtained for the translational and rotational dynamical properties of water confined at stacked phospholipid bilayers, we analyze the HB network formed by the water molecules. In fact, the dynamic and thermodynamic activity in liquid water is determined by the breaking and formation of the HBs [6,19,20,21,22,23]. Here, we adopt the widely employed geometric definition of the HB. We consider two molecules that are H-bonded when the distance between donor and acceptor oxygen atoms satisfies $d_{OO}<3.5$ Å, and the angle formed by the OH bond of the donor molecule with the OO direction is $\hat{\mathrm{HOO}}<30^{\circ }$. We consider HBs formed among water molecules and also among water and phosphate or ester groups of the DMPC phospholipid [12].

**Figure 4:**
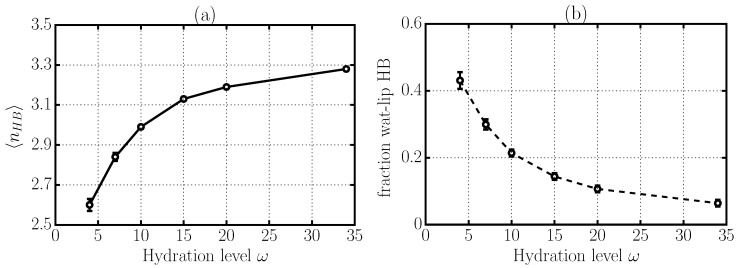
Changes in HB structure with hydration level *ω*. (**a**) average number $\langle n_{HB}\rangle$ of total HBs formed by each water molecule confined in stacked phospholipid membranes; (**b**) fraction of HBs between water molecules and lipid groups.

We find that the average number $\langle n_{HB}\rangle$ of HBs formed by each water molecule decreases significantly, from 3.3 to 2.6, as we reduce the membrane hydration *ω* (Figure 4a). Thus, at low *ω*, each water molecule has fewer H-bonds than at high *ω*. In addition, the percentage of HBs formed between water and lipids, with respect to the total number of HBs, increases from <10% at ω=34 to ≃45% at ω=4 (Figure 4b). Hence, at low *ω*, almost half of the water HBs are directly on the membrane. Overall, these findings suggest that at low *ω*, water is less bonded but more adsorbed to the membrane.

**Figure 5:**
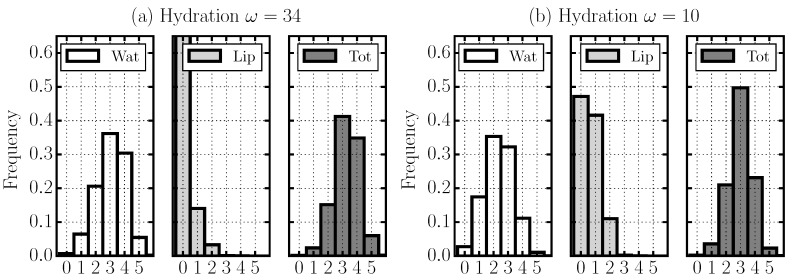

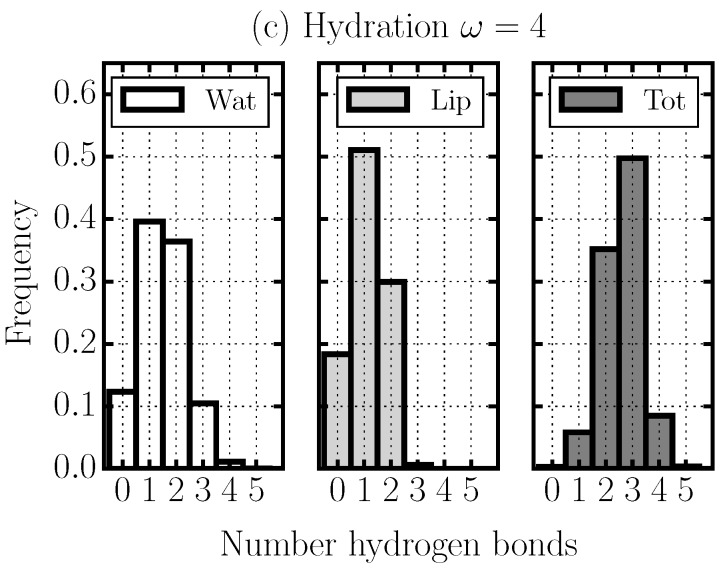
Normalized distributions of the number of HBs per water molecule in stacked membranes for (**a**) ω=34; (**b**) ω=10; and (**c**) ω=4. At each hydration level, we present the normalized distribution of water molecules forming a given number of HBs ≤5 with other water molecules (**left**), with lipid groups (**center**), and the normalized distribution of the sum of the two kinds of HBs (**right**).

To better understand how these results are related to the water slowdown, we perform a detailed analysis of the HB network for different values of *ω*. In particular, we calculate the distributions of the water–water HBs, the water–lipid HBs, and the sum of all them for decreasing *ω* (Figure 5).

**Figure 6:**
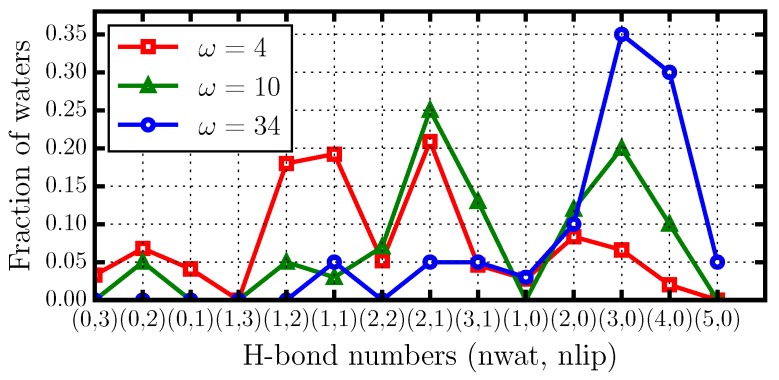
Fraction of water molecules forming $n_{\mathrm{wat}}$ HBs with other water molecules, and, at the same time, $n_{\mathrm{lip}}$ HBs with lipids, indicated along the *x*-axis as $\left(n_{\mathrm{wat}},n_{\mathrm{lip}}\right)$, for different hydration levels. At high hydration (ω=34, **blue** circles) the HBs are mainly among water molecules. At medium hydration (ω=10, **green** triangles) the most likely structures have $n_{\mathrm{wat}}=2$ and $n_{\mathrm{lip}}=1$, but still many water molecules have no HBs with lipids. At low hydration (ω=4, **red** squares), in general, the water HBs are involving at least one lipid and one or two more water molecules.

For the completely hydrated membrane (ω=34), we find that a water molecule forms preferentially three or four HBs (Figure 5a). Almost all of these HBs are between water molecules and very few involve lipids. This result can be verified by calculating the probability distribution that a water molecule forms $n_{\mathrm{wat}}$ HBs with other water molecules and at the same time $n_{\mathrm{lip}}$ HBs with lipids in the membrane (Figure 6).

For a membrane with intermediate hydration (ω=10), we find that water H-bonding decreases, with 3±1 HBs per water molecule (Figure 5b). In this case, water molecules preferentially form 1 HB with lipids and 2 HBs with other water molecules, but the probability of having only water–water HBs is high (Figure 6).

Finally, for the least hydrated membrane (ω=4), we find that a water molecule forms preferentially only two or 3 HBs, and it thus on average has fewer HBs than when *ω* is higher (Figure 5c). Our analysis reveals, however, that, under these conditions, there is a high probability that one or two of these HBs are with lipids , and, at the same time, with at least one other water molecule. Thus, the majority of the water–water HBs in the system bridge between water–lipids HBs. In addition, the probability that a water molecule is not bonded with at least one lipid is very small (Figure 5).

### 2.5. Hydrogen Bond Dynamics

**Figure 7:**
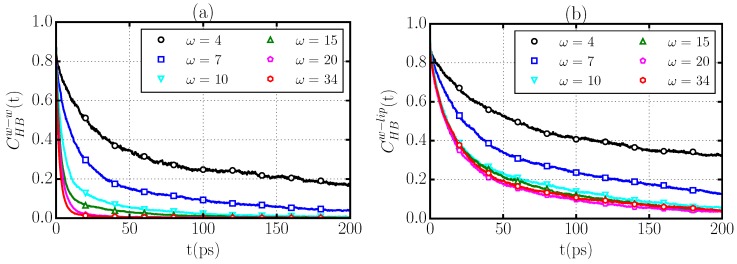
Relaxation of the time correlation functions $C_{HB}^{w-w}\left(t\right)$ (**a**) and $C_{HB}^{w-l}\left(t\right)$ (**b**) for stacked phospholipid membranes with hydration levels ω=4 (**black** circles), ω=7 (**blue** squares), ω=10 (**cyan** triangles down), ω=15 (**green** triangles up), ω=20 (**pink** pentagons), ω=34 (**red** hexagons).

To better relate our water HB structure analysis to the dynamics of confined water in stacked membranes, we investigate the time evolution of the HB network. We calculate the time correlation functions

(6) $$C_{HB}^{w-\alpha }\left(t\right)\equiv \frac{\langle n^{w-\alpha }\left(t\right)n^{w-\alpha }\left(0\right)\rangle }{\langle n^{w-\alpha }\left(0\right)\rangle }\;,$$

where $n^{w-\alpha }\left(t\right)$ is one when at time *t* a given water forms a HB with another water (α=w) or a lipid (α=l), and is zero otherwise. The brackets 〈...〉 indicate averaging over all water–water or water–lipid pairs and over multiple time origins. $C_{HB}^{w-\alpha }\left(t\right)$ is the measure of the probability that a HB at time 0 remains intact at a later time *t* (Figure 7). To quantify the relaxation of the correlation functions $C_{HB}^{w-\alpha }\left(t\right)$, we define the relaxation times

(7) $$\tau_{HB}^{w-\alpha }\equiv \int_{0}^{\infty }C_{HB}^{w-\alpha }\left(t\right)dt,\;$$

which are computed directly from the MD simulation trajectories.

We find that the relaxation of the water–water HB correlation function $C_{HB}^{w-w}\left(t\right)$ slows down dramatically as the hydration level of the membrane decreases (Figure 7a). In fact, the relaxation times of the water–water HB network (Table 3) increase monotonically from $\tau_{HB}^{w-w}=4.0$ ps for the completely hydrated membrane (ω=34) to $\tau_{HB}^{w-w}=110$ ps for ω=4. This behavior indicates that the water–water HBs in proximity to phospholipids, *i.e.*, at low *ω*, are significantly more stable than those formed in bulk, with correlation times that are ≃40 times longer than in bulk water. This result can be attributed at least to two causes:

1. the formation of water–water HBs that are in turn bonded through long-lived HBs to the phospholipid (Figure 7b), and
2. the decreased water density at the membrane interface ${\bar{\rho }}_{w}$ (Table 3), which hinders the H-bonding switch between water molecules [14,21].

However, we find that the water–water HBs are faster than the water–lipid HBs at any *ω*. In particular, $C_{HB}^{w-l}\left(t\right)$ exhibits a much slower relaxation than $C_{HB}^{w-w}\left(t\right)$ for all cases (Figure 7b). We quantify the effect by calculating the slowing-down factor $\kappa \equiv \tau_{HB}^{w-l}/\tau_{HB}^{w-w}$, which measures the relative dynamics of water–lipid and water–water HBs. We find that *κ* changes from ≃10 at complete hydration to ≃2 at low hydration (Table 3), indicating that the robustness of the water–lipid HBs with respect to those among water molecules persists even at very low hydration.

We observe that the relaxation of water–lipid HBs is unaffected by increasing the level of membrane hydration above a threshold ω≥ 10 (Figure 7b), with relaxation times that reach a stable value $\tau_{HB}^{w-l}\approx$ 40 ps for ω≥ 10 (Table 3). This result suggests that for 7<ω<10, there is a saturation of the water–membrane interface where water molecules directly interact with lipid headgroups in agreement with X-ray scattering experiments [24]. As the hydration level is increased, this region of the interface is not modified.

### 2.6. Water Dynamics Determined by Distance to Membrane

The results presented thus far for the diffusion and rotational dynamics of water molecules confined in stacked membranes are averaged properties over all water molecules. These averages are experimentally accessible [7,10,17] but provide no further insight into the local interfacial mechanisms that govern the dynamics of confined water at different hydration levels. To achieve this, we perform a detailed analysis of our MD simulation results.

We first define the water–membrane distance in order to quantify the water–membrane interface. This definition is necessary because, within the relevant lengthscale of the problem, which corresponds to the size of a water molecule, the water–membrane interface is not flat and exhibits spatial inhomogeneities. In addition, the interface is soft and can be penetrated by water molecules, as observed in experiments [4] and numerical simulations [25,26].

**Figure 8:**
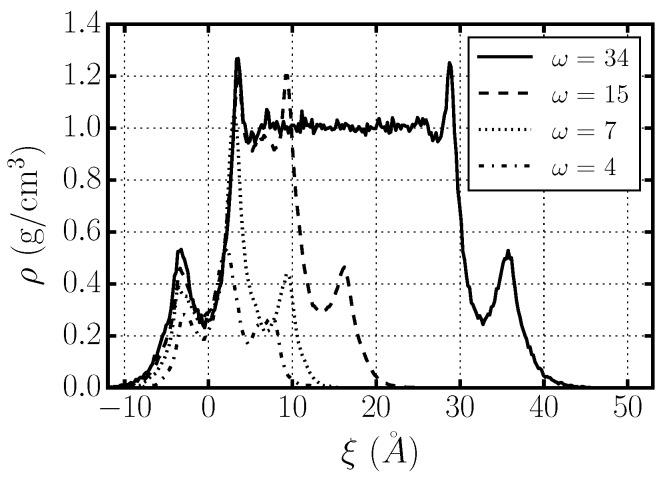
Density of water molecules confined between stacked DMPC bilayers with hydration level ω=4, 7, 15, 34, as a function of the membrane distance *ξ* with respect to one of the two confining membranes. We define three relevant regions: (i) interior of the membrane, with ξ<0; (ii) first hydration layer, with 0<ξ<5 Å; (iii) exterior of the membrane, with ξ>5 Å.

In particular, we follow Pandit *et al.* [3,25] when defining the distance from the membrane. For each MD snapshot, we perform a two-dimensional Voronoi tessellation of the average plane of the membrane (the xy-plane) using the phosphorous and nitrogen atoms of the phospholipid heads as centers of the Voronoi cells. To each water molecule, we assign a Voronoi cell by its projection in the xy-plane and a membrane distance $\xi \equiv z_{\mathrm{water}}-z_{\mathrm{Voronoy}}$, where $z_{\mathrm{water}}$ and $z_{\mathrm{Voronoy}}$ are the z-coordinates of the water molecule and its corresponding Voronoi cell, respectively. Finally, we define three different regions relevant to the water–membrane interface, (i) the interior of the membrane, with ξ<0; (ii) the first hydration layer, with 0<ξ<5 Å; and (iii) the exterior of the membrane, with ξ>5 Å (Figure 8).

For each of these three regions of a completely hydrated membrane (ω=34), we calculate the average diffusion coefficient $D_{∥}$ for translations parallel to the membrane xy-plane and the average characteristic time $\tau_{\mathrm{rot}}$ for dipolar rotational relaxation (Table 4). We find similar results (not shown) for all hydrations, including ω=4 and 7, but, in these cases, there is no exterior region.

Our calculations indicate that the dynamics of water in the interior of the membrane (ξ<0) are very sluggish. In particular, this water diffuses ≈30 times more slowly than bulk water, and has a rotational relaxation time ≈100 times longer than bulk water.

We find that water in the first hydration layer (0<ξ<5 Å) is also slower than bulk water, both in the translational diffusion time, ≈10 times slower than bulk water, and the rotational relaxation time, ≈10 times longer than bulk water. We interpret these results as a consequence of the strong interaction of water in the first hydration layer with the phospholipid membrane.

Finally, we observe that the dynamic behavior of water in the exterior region (ξ>5 Å) resembles that in bulk water, with both $D_{∥}$ and $\tau_{\mathrm{rot}}$ approaching the bulk water values. Nevertheless, both the translational and the rotational dynamics are slower than in bulk water, outside the error bars, indicating that the nearby membrane has a residual influence that extends beyond the first hydration layer.

These results provide a simple interpretation of how the overall dynamics of water confined in stacked phospholipid membranes depends on hydration: as the hydration increases, water first saturates the inner part of the membrane, next fills the layer in contact with the membrane (for ω≈10), and finally fills the space between the stacked membranes layer by layer (Figure 8). As a consequence, for ω≳10, as the hydration increases, the fraction of bulk-like water molecules increases and the overall water dynamics becomes faster.

## 3. Methods

We prepare six different systems of hydrated phospholipid bilayers with hydration levels (*i.e.*, water molecules per lipid) ω=4, 7, 10, 15, 20, and 34 (Figure 1). We examine a range that extends from the weakly hydrated systems probed in recent experiments [5,8,9,10] to a fully hydrated membrane (with hydration level ω=34), which has been thoroughly studied both experimentally and using computer simulations [12,16]. The bilayer is composed of 128 dimyristoylphosphatidylcholine (DMPC) lipids distributed in two leaflets. We apply periodic boundary conditions in all three dimensions, which allows us to describe a system of stacked bilayers with perfect periodicity along a direction perpendicular to the plane of the membrane.

We perform MD simulations using the NAMD 2.9 [27] package at a temperature of 303 K and an average pressure of 1 atm. We set the simulation time step to 1 fs. We describe the structure of phospholipids and their mutual interactions using the recently parameterized force field CHARMM36 [28,29], which is able to reproduce the area per lipid in excellent agreement with experimental data. The water model employed in our simulations, consistent with the parametrization of CHARMM36, is the modified TIP3P [30,31]. We cut off the van der Waals interactions at 12 Å with a smooth switching function starting at 10 Å. We compute the long-range electrostatic forces using the particle mesh Ewald method [32] with a grid space of ≈1 Å. We update the electrostatic interactions every 2 fs. After energy minimization, we equilibrate the hydrated phospholipid bilayers for 10 ns followed by a production run of 50 ns in the NPT ensemble at 1 atm. The simulations of bulk water were equilibrated in the NPT ensemble for 1 ns, followed by a production run in the NVT ensemble of 5 ns. In the simulations, we use a Langevin thermostat [33] with a damping coefficient of 0.1 ps${}^{-1}$ to control the temperature and a Nosé–Hoover Langevin barostat [34] with a piston oscillation time of 200 fs and a damping time of 100 fs to control the pressure.

The use of a Langevin thermostat as a temperature control algorithm in MD simulations has been shown to induce an artificial damping in the dynamical properties of the simulated system when strong couplings are used [35]. In the weak coupling regime (e.g., in the simulations reported in this work), it has been shown that the damping effect of the Langevin thermostat is negligible when studying hydrated membranes [36]. We have also performed simulations of 1981 bulk TIP3P water molecules in a cubic simulation box with periodic boundary conditions coupled to a Langevin thermostat that produces a diffusion coefficient (D=5.3±0.1 at *T* = 298 K) that agrees with the value reported in the literature for MD simulations of TIP3P water coupled to a Berendsen thermostat [37].

## 4. Conclusions

We investigate the dynamical properties of water confined in stacked DMPC phospholipid membranes with different hydration levels, ranging from poorly hydrated systems (with ω=4 water molecules per phospholipid corresponding to approximately one layer of water between the two membranes, with a membrane-to-membrane distance of h≲0.3 nm) to a completely hydrated membrane (with ω=34, with ∼10 confined water layers, h≲3 nm). We find that both the translational diffusion and the reorientation dynamics of water slow dramatically when the hydration level is reduced. In particular, we calculate that the diffusion coefficient of water parallel to the plane of the membrane monotonically decreases from $D_{∥}=3.4$ nm${}^{2}$/ns in a completely hydrated membrane to $D_{∥}=0.13$ nm${}^{2}$/ns in the lowest hydrated case. In addition, we find a similar behavior in the water reorientation dynamics, for which the characteristic relaxation times increase monotonically from $\tau_{\mathrm{rot}}=12.4$ ps at ω=34 to $\tau_{\mathrm{rot}}=290$ ps at ω=4.

These results can be partially attributed to the heterogeneity of the interface, which generates a rugged energy landscape for the confined water, consistent with recent theoretical work [18], and they are in agreement with experiments performed under similar conditions. In particular, the rotational dynamics can be divided, as suggested in Ref. [10], into three contributions from three different populations of water with different relaxation times, one *fast*, one *bulk-like*, and one *irrotational*. However, we show that the resulting fitting parameters do not indicate a regular behavior and suggest that this interpretation might be incomplete.

We thus offer an interpretation in terms of HB structure and dynamics. We find that the most likely value for the number of HBs per water molecule is $n_{HB}=3$ for all the considered hydrations. However, the secondary peaks of the overall distribution of $n_{HB}$ move toward lower values as *ω* is decreased, implying a decrease in the average $\langle n_{HB}\rangle$. This decrease leads to an increase in the probability that a water molecule will have a single HB, which might represent the *fast* reorienting water observed experimentally [8,10].

We further analyze the HB dynamics and find that the translational and rotational slowdown in water confined between stacked DMPC phospholipid bilayers when the hydration level is lowered can be attributed to a combination of several factors:

(i) the slow dynamics of water–lipid HBs,
(ii) the higher proportion of water–lipid HBs at low hydrations,
(iii) the slowdown of water–water HBs that bridge between persistent water–lipid HBs, and
(iv) the slowdown of HB-switching due to the decrease of water density.

Finally, we perform a local analysis of the dynamics at the interface. We find that water molecules in the interior of the membrane are up to two orders of magnitude slower than bulk water. Water in the first hydration layer is one order of magnitude slower than bulk water, indicating that the slowdown effect of the membrane decreases rapidly as the distance from the interface is increased. Yet we find a residual influence of the membrane that slows down the water dynamics beyond the first hydration layer.

This layer-by-layer analysis suggests that there are three components contributing to the averaged water dynamics: (i) a bulk-like component from water away from the membrane; (ii) a very sluggish component (both translationally and rotationally) from the water trapped inside the membrane; and (iii) a slow component at the interface between (i) and (ii). Although this lays aside any interpretation that utilizes a fast component in water relaxation, our calculation of average quantities cannot exclude the presence of heterogeneities, such as those associated with water molecules with a single HB as discussed above.

In conclusion, although our analysis clearly shows that the formation of long-duration, slow-relaxing water HBs with lipids at the interface with the membrane causes the dynamics of water confined between membranes to slow down, and we cannot exclude the possibility that there is a fast component that contributes to the dynamics of hydration water, and more detailed study beyond the scope of this work is needed. Our results and their possible extension contribute to our understanding of the properties of biological membranes.

## Figures and Tables

**Table 1:** Rotational relaxation time as calculated in Equation (3) for the dipolar correlation function.

Hydration (*ω*)	4	7	10	15	20	34
$\tau_{\mathrm{rot}}$ (ps)	290 ± 10	99 ± 4	45.6 ± 0.5	29.0 ± 0.5	21.7 ± 0.3	12.4 ± 0.3

**Table 2:** The five parameters of the fits for the rotational dipolar correlation function $C_{\mathrm{sim}}^{\mathrm{rot}}\left(t,\omega \right)$ resulting from MD simulations of stacked membranes with different hydration level *ω*.

*ω*	$\tau_{\mathrm{fast}}$ (ps)	$\tau_{\mathrm{bulk}}$ (ps)	$\tau_{\mathrm{irr}}$ (ps)	$f_{\mathrm{fast}}$	$f_{\mathrm{bulk}}$	$f_{\mathrm{irr}}=1-f_{\mathrm{fast}}-f_{\mathrm{bulk}}$
4	0.53	5.1	321	0.10 ±0.02	0.10 ±0.02	0.8 ±0.03
7	0.48	9.8	168	0.11 ±0.03	0.31 ±0.01	0.58 ±0.04
10	0.5	10.3	154	0.09 ±0.03	0.62 ±0.02	0.29 ±0.04
15	0.44	4.8	121	0.06 ±0.02	0.70 ±0.01	0.24 ±0.02
20	0.48	5.2	130	0.05 ±0.03	0.75 ±0.01	0.2 ±0.04
34	0.47	3.2	117	0.04 ±0.02	0.85 ±0.02	0.11 ±0.03

**Table 3:** Relaxation times of the correlation functions $C_{HB}^{w-\alpha }\left(t\right)$, $\tau_{HB}^{w-\alpha }$, with α=w,l, for stacked phospholipid membranes with hydration level *ω*. For each system, we provide the value of the average water density, given by ${\bar{\rho }}_{w}\equiv N_{w}/\left(A_{XY}\times d\right)$, where $N_{w}$ is the number of water molecules in the system, $A_{XY}$ is the area of the membrane, and *d* is the width of the water–membrane interface. We also explicitly indicate the slowing-down factor *κ* of the w-l with respect to the w-w HBs.

Hydration (*ω*)	$\tau_{\mathrm{HB}}^{w-w}$ (ps)	$\tau_{\mathrm{HB}}^{w-l}$ (ps)	${\bar{\rho }}_{w}$ (g/cm${}^{3}$)	$\kappa \equiv \tau_{\mathrm{HB}}^{w-l}/\tau_{\mathrm{HB}}^{w-w}$
4	110 ±10	242 ±10	0.21	2.2
7	31 ±5	80 ±10	0.30	2.6
10	12 ±2	44 ±5	0.37	3.7
15	7.1 ±0.5	38 ±5	0.45	5.3
20	5.1 ±0.5	35 ±5	0.52	6.7
34	4.0 ±0.5	38 ±5	0.65	9.5
bulk	1.8 ±0.1	–	0.99	–

**Table 4:** Diffusion coefficient $D_{∥}$ in the xy-plane parallel to the membrane and rotational dipolar relaxation time $\tau_{\mathrm{rot}}$, both averaged over the water molecules in different regions of the water–membrane interface at ω=34, compared with the values for bulk water at same thermodynamic conditions (P=1 atm, T=303 K).

Layer	D${}_{∥}$ (nm${}^{2}$/ns)	$\tau_{\mathrm{rot}}$ (ps)
Interior	0.19 ± 0.05	270 ±10
1st hydration layer	0.81 ±0.03	37 ± 5
Exterior	4.10 ±0.05	3.3 ± 0.2
Bulk	5.5 ±0.1	2.4 ± 0.1

## References

[1] Hamley I.W. (2007). Introduction to Soft Matter.

[2] Nagle J.F., Tristram-Nagle S. (2000). Structure of lipid bilayers. Biophys. Acta.

[3] Berkowitz M.L., Bostick D.L., Pandit S. (2006). Aqueous solutions next to phospholipid membrane surfaces: Insights from simulations. Chem. Rev..

[4] Fitter J., Lechner R.E., Dencher N.A. (1999). Interactions of hydration water and biological membranes studied by neutron scattering. J. Phys. Chem. B.

[5] Trapp M., Gutberlet T., Juranyi F., Unruh T., Demé B., Tehei M., Peters J. (2010). Hydration dependent studies of highly aligned multilayer lipid membranes by neutron scattering. J. Chem. Phys..

[6] Mazza M.G., Stokely K., Pagnotta S.E., Bruni F., Stanley H.E., Franzese G. (2011). More than one dynamic crossover in protein hydration water. Proc. Natl. Acad. Sci. USA.

[7] Wassall S.R. (1996). Pulsed field gradient-spin echo NMR studies of water diffusion in a phospholipid model membrane. Biophys. J..

[8] Volkov V.V., Palmer D.J., Righini R. (2007). Distinct Water species confined at the interface of a phospholipid membrane. Phys. Rev. Lett..

[9] Zhao W., Moilanen D.E., Fenn E.E., Fayer M.D. (2008). Water at the surfaces of aligned phospholipid multibilayer model membranes probed with ultrafast vibrational spectroscopy. J. Am. Chem. Soc..

[10] Tielrooij K.J., Paparo D., Piatkowski L., Bakker H.J., Bonn M. (2009). Dielectric relaxation dynamics of water in model membranes probed by Terahertz spectroscopy. Biophys. J..

[11] Róg T., Murzyn K., Pasenkiewicz-Gierula M. (2002). The dynamics of water at the phospholipid bilayer surface: A molecular dynamics simulation study. Chem. Phys. Lett..

[12] Bhide S.Y., Berkowitz M.L. (2005). Structure and dynamics of water at the interface with phospholipid bilayers. J. Chem. Phys..

[13] Von Hansen Y., Gekle S., Netz R.R. (2013). Anomalous anisotropic diffusion dynamics of hydration water at lipid membranes. Phys. Rev. Lett..

[14] Zhang Z., Berkowitz M.L. (2009). Orientational dynamics of water in phospholipid bilayers with different hydration levels. J. Phys. Chem. B.

[15] Gruenbaum S., Skinner J. (2011). Vibrational spectroscopy of water in hydrated lipid multi-bilayers. I. Infrared spectra and ultrafast pump-probe observables. J. Chem. Phys..

[16] Nagle J.F., Zhang R., Tristram-Nagle S., Petrache H.I., Suter R.M. (1996). X-ray structure determination of fully hydrated L alpha phase dipalmitoylphosphatidylcholine bilayers. Biophys. J..

[17] Rudakova M., Filippov A., Skirda V. (2004). Water diffusivity in model biological membranes. Appl. Magn. Reson..

[18] Seki K., Bagchi K., Bagchi B. (2016). Anomalous dimensionality dependence of diffusion in a rugged energy landscape: How pathological is one dimension?. arXiv Preprint.

[19] Franzese G., Stanley H.E. (2002). Liquid-liquid critical point in a Hamiltonian model for water: Analytic solution. J. Phys. Condens. Matter.

[20] Kumar P., Franzese G., Buldyrev S.V., Stanley H.E. (2006). Molecular dynamics study of orientational cooperativity in water. Phys. Rev. E.

[21] Laage D., Hynes J.T. (2006). A molecular jump mechanism of water reorientation. Science.

[22] Stokely K., Mazza M.G., Stanley H.E., Franzese G. (2010). Effect of hydrogen bond cooperativity on the behavior of water. Proc. Natl. Acad. Sci. USA.

[23] De los Santos F., Franzese G. (2012). Relations between the diffusion anomaly and cooperative rearranging regions in a hydrophobically nanoconfined water monolayer. Phys. Rev. E.

[24] Kučerka N., Liu Y., Chu N., Petrache H.I., Tristram-Nagle S., Nagle J.F. (2005). Structure of fully hydrated fluid phase DMPC and DLPC lipid bilayers using X-ray scattering from oriented multilamellar arrays and from unilamellar vesicles. Biophys. J..

[25] Pandit S.A., Bostick D., Berkowitz M.L. (2003). An algorithm to describe molecular scale rugged surfaces and its application to the study of a water/lipid bilayer interface. J. Chem. Phys..

[26] Lopez C.F., Nielsen S.O., Klein M.L., Moore P.B. (2004). Hydrogen bonding structure and dynamics of water at the dimyristoylphosphatidylcholine lipid bilayer surface from a molecular dynamics simulation. J. Phys. Chem. B.

[27] Phillips J.C., Braun R., Wang W., Gumbart J., Tajkhorshid E., Villa E., Chipot C., Skeel R.D., Kalé L., Schulten K. (2005). Scalable molecular dynamics with NAMD. J. Comput. Chem..

[28] Klauda J.B., Venable R.M., Freites J.A., O’Connor J.W., Tobias D.J., Mondragon-Ramirez C., Vorobyov I., MacKerell A.D., Pastor R.W. (2010). Update of the CHARMM all-atom additive force field for lipids: Validation on six lipid types. J. Phys. Chem. B.

[29] Lim J.B., Rogaski B., Klauda J.B. (2012). Update of the cholesterol force field parameters in CHARMM. J. Phys. Chem. B.

[30] Jorgensen W.L., Chandrasekhar J., Madura J.D., Impey R.W., Klein M.L. (1983). Comparison of simple potential functions for simulating liquid water. J. Chem. Phys..

[31] MacKerell A.D., Bashford D., Bellott M.L.D.R., Dunbrack R.L., Evanseck J.D., Field M.J., Fischer S., Gao J., Guo H., Ha S. (1998). All-atom empirical potential for molecular modeling and dynamics studies of proteins. J. Phys. Chem. B.

[32] Essmann U., Perera L., Berkowitz M.L., Darden T., Lee H., Pedersen L.G. (1995). A smooth particle mesh Ewald method. J. Chem. Phys..

[33] Berendsen H.J.C., Postma J.P.M., Gunsteren W., DiNola A., Haak J.R. (1984). Molecular dynamics with coupling to an external bath. J. Phys. Chem..

[34] Feller S.E., Zhang Y., Pastor R.W., Brooks B.R. (1995). Constant pressure molecular dynamics simulation: The Langevin piston method. J. Phys. Chem..

[35] Basconi J.E., Shirts M.R. (2013). Effects of temperature control algorithms on transport properties and kinetics in molecular dynamics simulations. J. Chem. Theory Comput..

[36] Aksimentiev A., Schulten K. (2005). Imaging *α*-hemolysin with molecular dynamics: Ionic conductance, osmotic permeability, and the electrostatic potential map. Biophys. J..

[37] Mahoney M.W., Jorgensen W.L. (2001). Diffusion constant of the TIP5P model of liquid water. J. Chem. Phys..

